# Supplementary material for: A reconfigurable and conformal liquid sensor for ambulatory cardiac monitoring
Source: Nat Commun. 2024 Oct 1;15:8492. doi: 10.1038/s41467-024-52462-8 (PMC11445489; doi:10.1038/s41467-024-52462-8)
Supplement: Supplementary file 1 — Supplementary Information [file 41467_2024_52462_MOESM1_ESM.pdf]

Supplementary Information

A reconfigurable and conformal liquid sensor for ambulatory cardiac monitoring

Xun Zhao[1,#], Yihao Zhou[1,#], William Kwak[1], Aaron Li[1], Shaolei Wang[1], Marklin Dallenger[1], Songyue Chen[1], Yuqi Zhang[1], Allison Lium[1], Jun Chen[1,*]

[1]Department of Bioengineering, University of California, Los Angeles, Los Angeles, CA 90095, USA

[#]These authors contributed equally to this work.

* Correspondence to jun.chen@ucla.edu (J.C.)

This PDF file includes:

Supplementary Figures 1-25

Supplementary Notes 1-5

Supplementary Table 1

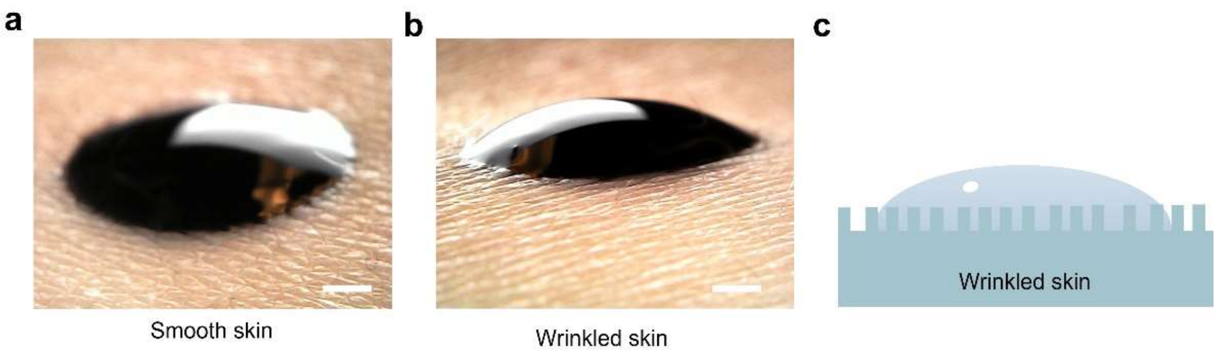

Supplementary Figure 1. Picture of liquid cardiac sensor application. a, Application of liquid cardiac sensor on smooth skin. Scale bar, 5 mm. **b**, Application of liquid cardiac sensor on wrinkled skin. Scale bar, 5 mm. **c**, Diagram of the wrinkled skin and the liquid cardiac device.

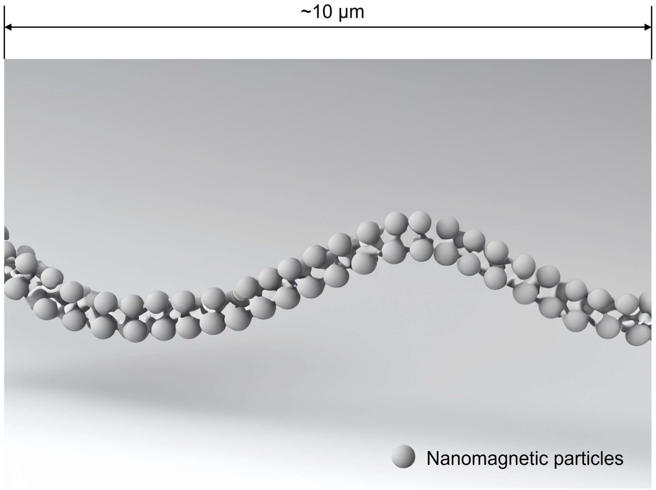

Supplementary Figure 2. 3D diagram of ORM network. 3D structure of ORM nanostructure formed in a carrier fluid.

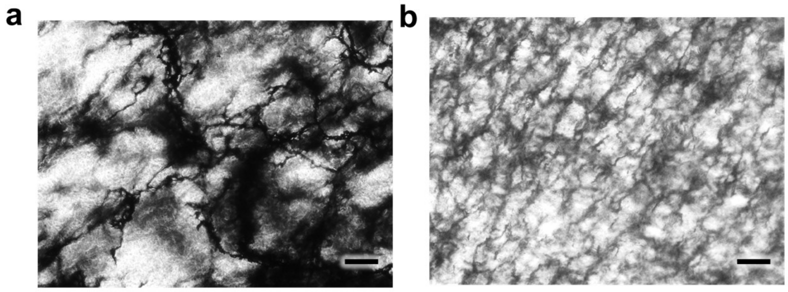

Supplementary Figure 3. Microscopic image of 3D ORM network. a-b, Microscopic images of ORM network in the liquid cardiac sensor. Scale bars, 100 μm.

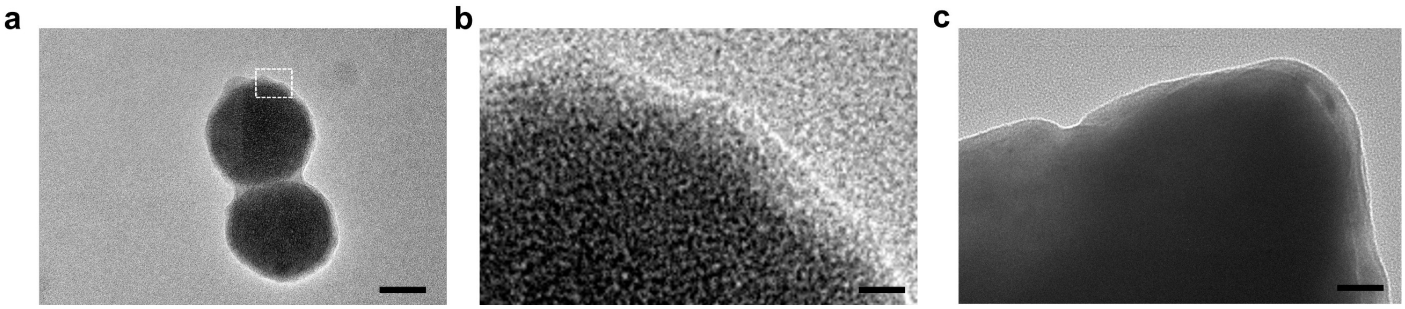

Supplementary Figure 4. TEM image of magnetic nanoparticles. a, Scale bar, 100 nm. **b,** Magnified image of the white box in 4a. Scale bar, 10 nm. **c,** Scale bar, 20 nm. All images were conducted following three independent experiments. Those images yield similar results in different independent experiments. Typical images were shown in the Figure.

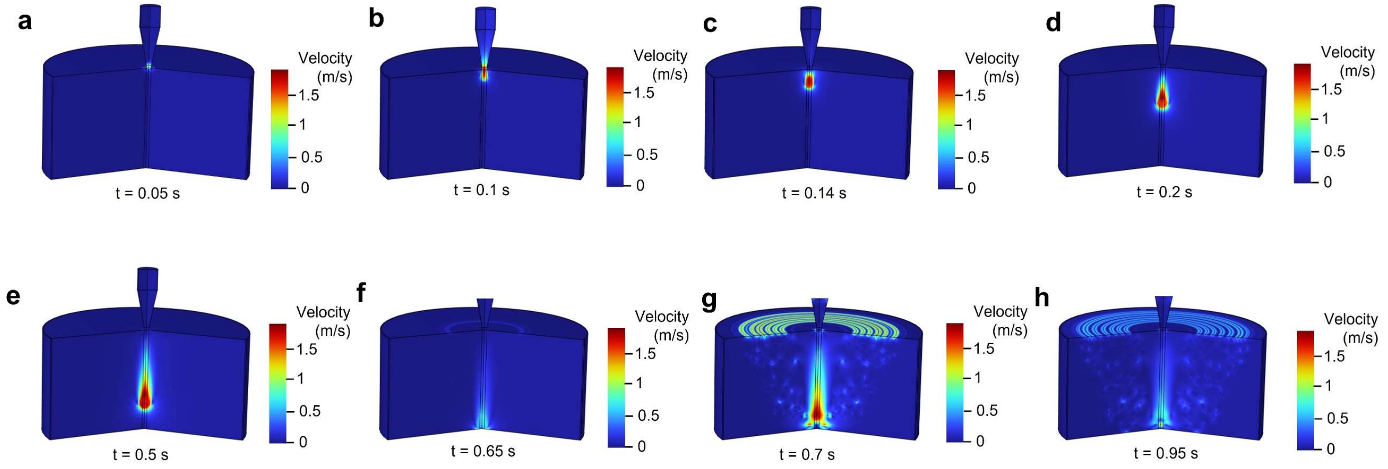

Supplementary Figure 5. Finite element analysis was constructed to simulate the injection process. Results show the relationship between time and droplet velocity. **a**, t = 0.05 s, **b**, t = 0.1 s, **c**, t = 0.14 s, **d**, t = 0.2 s, **e**, t = 0.5 s, **f**, t = 0.65 s, **g**, t = 0.7 s, **h**, t = 0.95 s.

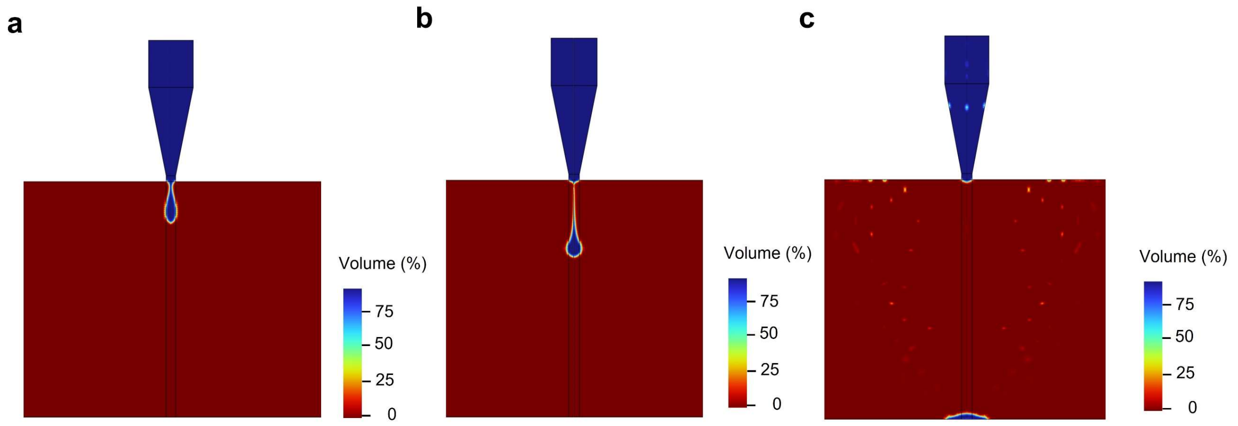

Supplementary Figure 6. Finite element analysis was constructed to simulate the injection process. Results show the relationship between time and droplet volume. **a,** t = 0.1 s. **b,** t = 0.2 s. **c,** t = 0.65 s.

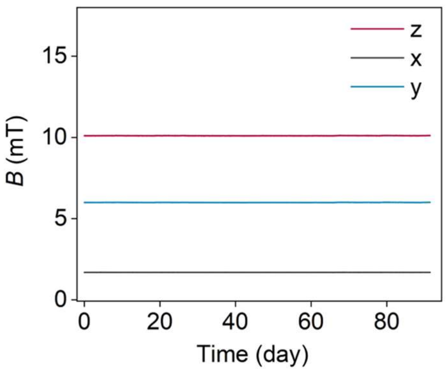

Supplementary Figure 7. Testing the magnetic field of the PFM for three months.

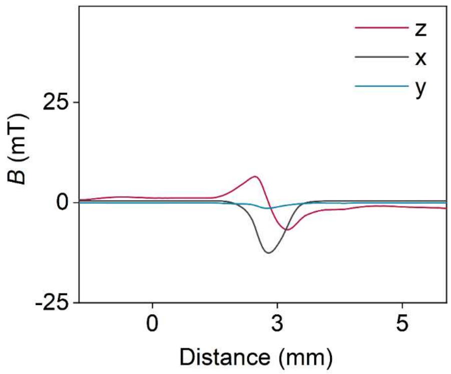

Supplementary Figure 8. Scanning the magnetic field of the PFM in three directions.

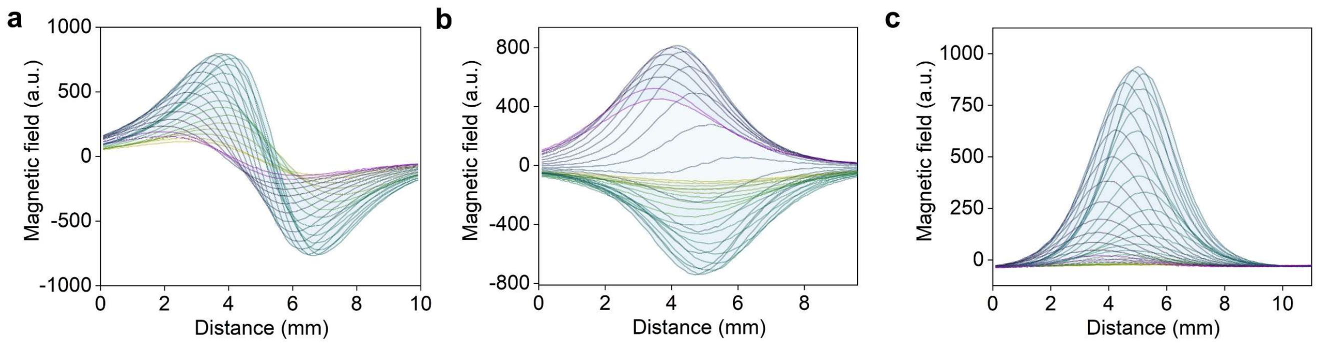

Supplementary Figure 9. a-c, Magnetic field output measurements in (**a**) x-, (**b**) y-, and (**c**) z-directions from PFM droplet of 10 mm in length across multiple trials.

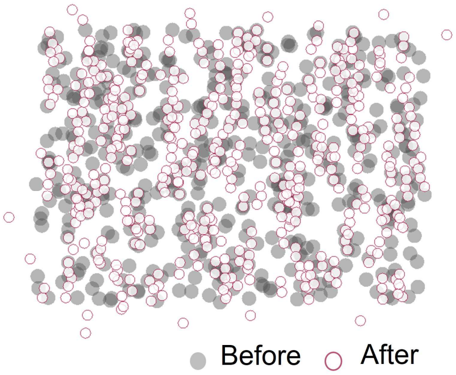

Supplementary Figure 10. Monte Carlo simulation to replicate the formation of the network structure.

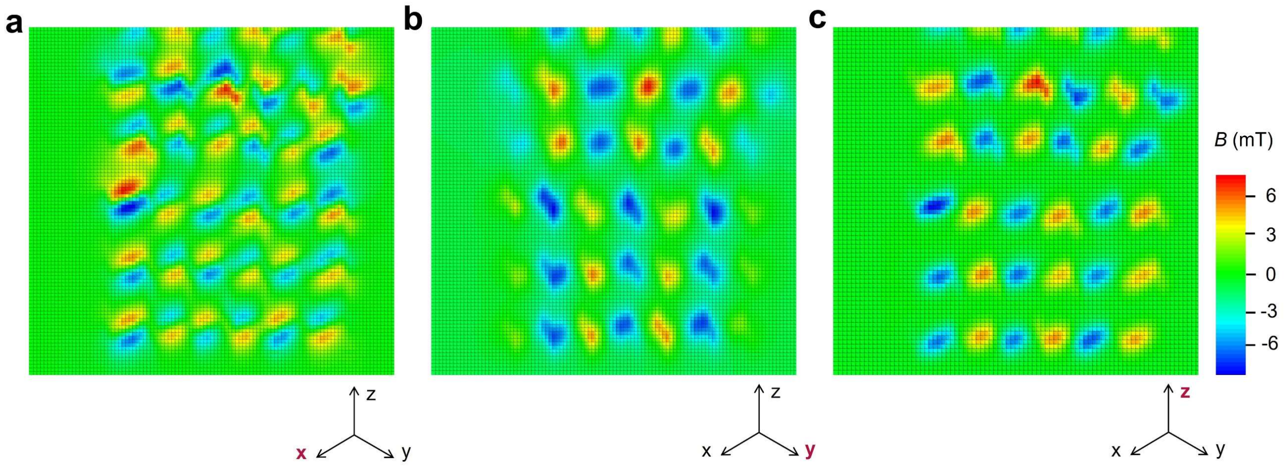

Supplementary Figure 11. Magnetic field mapping of a PFM droplet array. a, Magnetic field map in x-axis **b,** Magnetic field map in y-axis **c,** Magnetic field map in z-axis across 12 PFM droplets arranged in a grid pattern. Measured magnetic fields of the PFM droplet array are in the range of 12mT (red) to -12mT (blue).

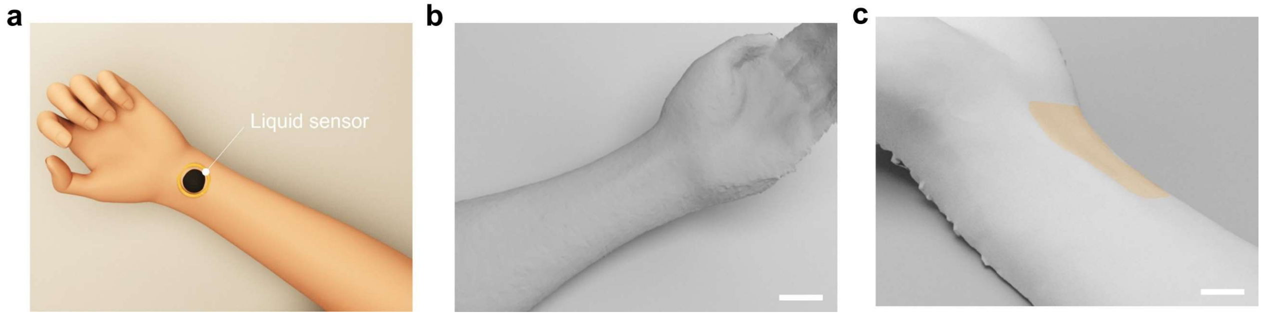

Supplementary Figure 12. Curve of the wrist from 3D scanning. a, Illustration showing the generation of magnetic field variations by the passage of blood flow. **b-c,** Illustration showing the 3d scanned surface. The yellow highlight is the wrist. Scale bars, 10 mm.

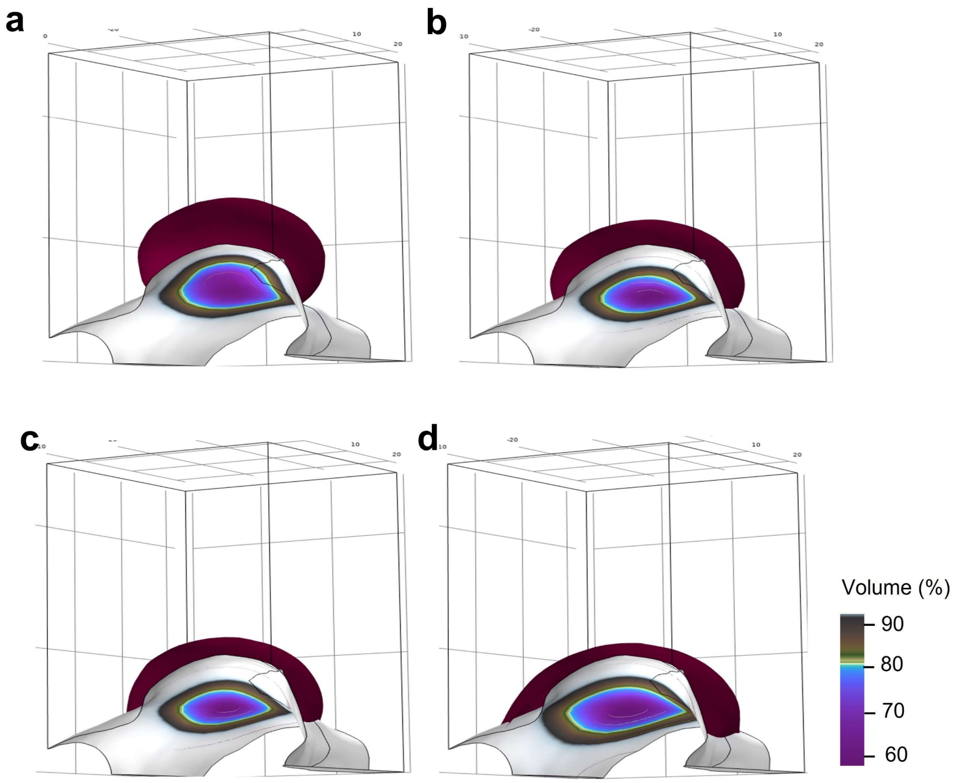

Supplementary Figure 13. A finite element simulation showing the liquid sensor was released on the wrist.

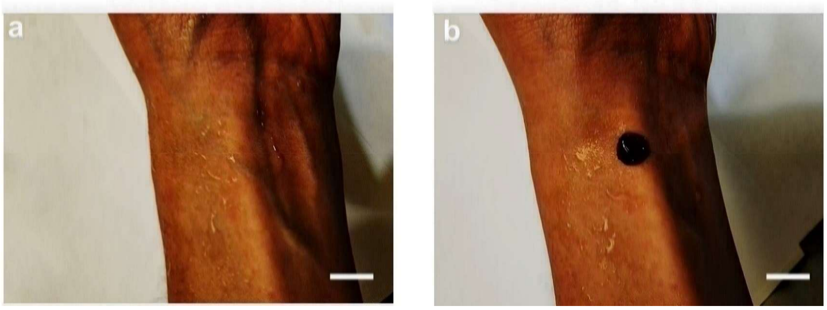

Supplementary Figure 14. Pictures of the liquid sensor attached to wet skin. a, Wet skin. **b**, Liquid sensor on wet skin. Scale bars, 8 mm.

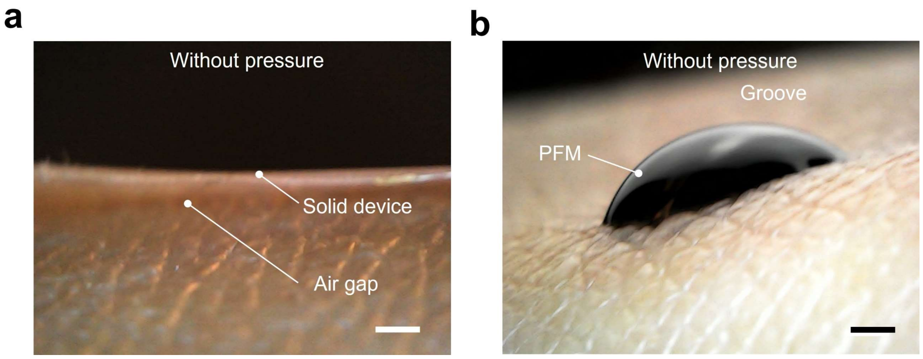

Supplementary Figure 15. Comparison between current solid materials and liquid materials. a, Zoomed-in image of a solid device on human skin. Scale bar, 250 µm. **b**, Zoomed-in image of the ferromagnetic droplet on human skin. Scale bar, 250 µm. All images were conducted following three independent experiments. Those images yield similar results in different independent experiments. Typical images were shown in the Figure.

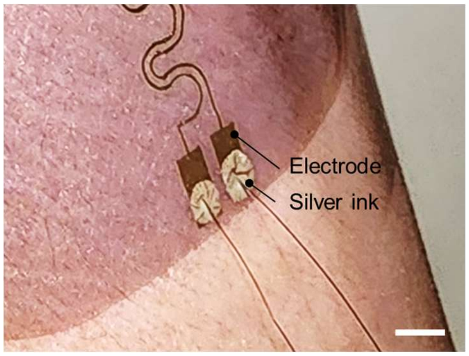

Supplementary Figure 16. Picture showing the connection between the wire and electrodes. Scale bar, 2 mm. All images were conducted following three independent experiments. Those images yield similar results in different independent experiments. Typical images were shown in the Figure.

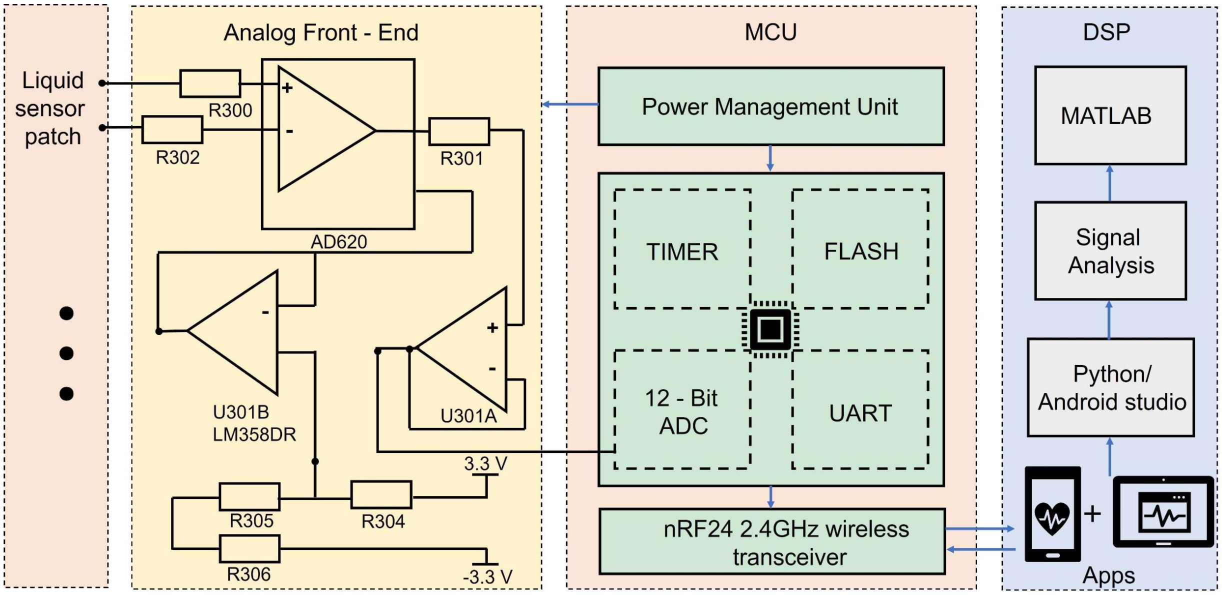

Supplementary Figure 17. Circuit diagram of biosensing board for signal collection.

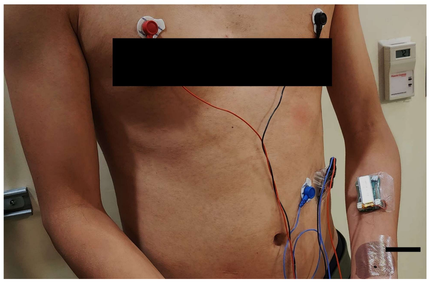

Supplementary Figure 18. Experimental setup. Experimental setup to measure the liquid sensor and electrocardiogram (ECG). Scale bar, 5 cm.

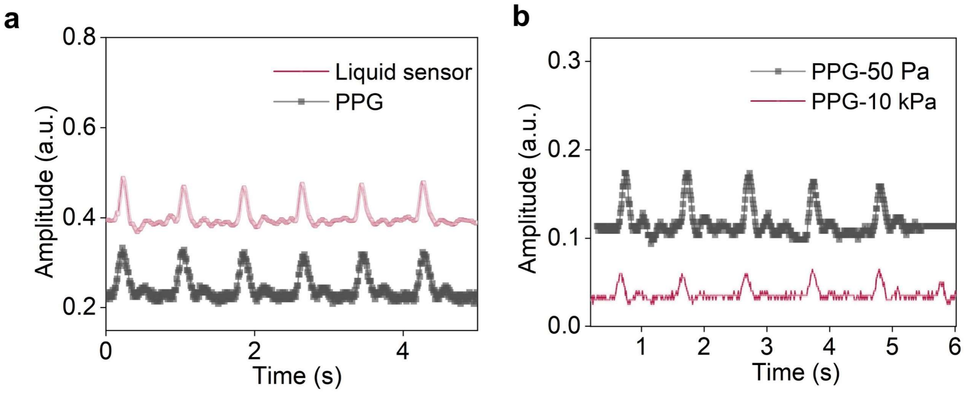

Supplementary Figure 19. Comparison between the liquid sensor and a gold-standard PPG pulse wave monitoring device. a, Comparison between liquid sensor and PPG pulse wave monitoring device. **b,** PPG pulse wave monitoring device under stress of 50 Pa and 10 kPa.

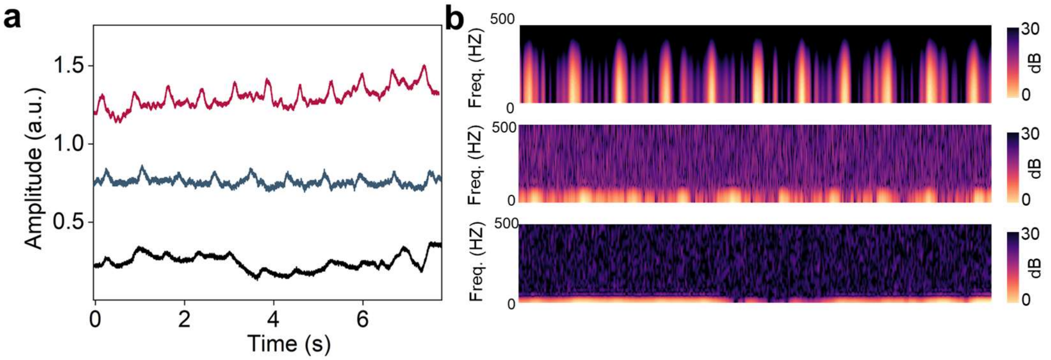

Supplementary Figure 20. Pulse wave measured from solid-based sensor. **a**, Black line is without pressure (zero pressure), blue line is 12 Pa, red line is 10 kPa. **b**, Measured pulse wave from solid based sensor in different conditions. Short-time Fourier transform of each trial. It shows that increased pressure gives a better signal.

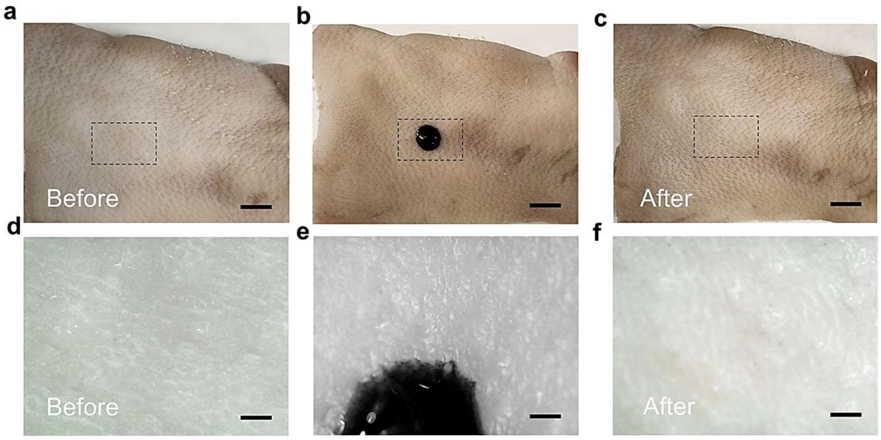

Supplementary Figure 21. The cleaning process of the liquid cardiac sensor. a-c, The PFM was added and removed on the skin. Scale bars, 1 cm. **d-f,** Microscope image of the skin when PFM was added and removed. Scale bars, 250 µm. All images were conducted following three independent experiments. Those images yield similar results in different independent experiments. Typical images were shown in the Figure.

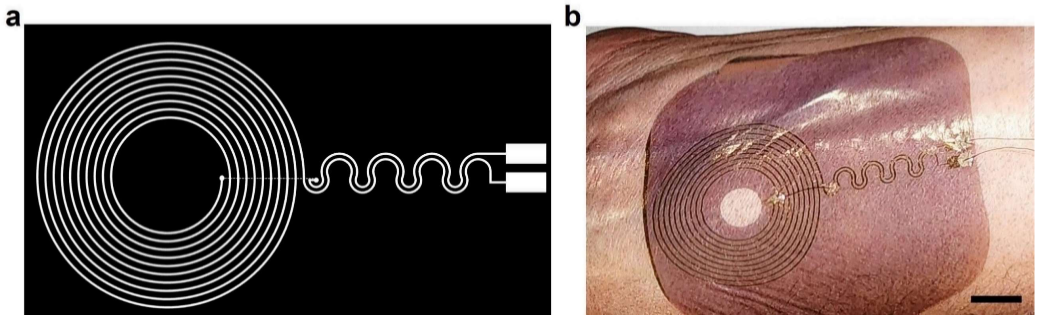

Supplementary Figure 22. Conformal pick-up coil on the skin. a, Design diagram of the conformal pick-up coil. **b**, Picture of the conformal pick-up coil. Scale bar, 1 cm.

Supplementary Note 1. Setups for the Monte Carlo simulation.

We performed a 3-dimensional Monte Carlo simulation to help study and understand the formation of the ORM network in the PFM liquid biosensors. To enhance the simulation efficiency, we have used nanomagnet clusters as the single element in the Monte Carlo simulation. A total of 500 clusters were employed in the study. All the clusters are considered as dipolar hard spheres with a radius of 1.5 μm and the system's total energy in terms of dipole-dipole interaction and truncated Lennard-Jones potential can be expressed as below,

$$U = 4\varepsilon \sum_{i,j,\ i\neq j} \left\{ (\frac{2R}{r_{ij}})^{12} - (\frac{2R}{r_{ij}})^{6} - (\frac{2R}{r_c})^{12} + (\frac{2R}{r_c})^{6} \right\}$$

$$- \frac{2}{3}\pi R^3 \mu_0 \sum_{i} \left\{ \frac{1}{\chi}\mathbf{M}_{\mathrm{i}}{}^2 + R^3 \sum_{j,\ j\neq i} \left[\frac{(\mathbf{M}_{\mathrm{i}} \cdot \mathbf{r}_{\mathrm{ij}})(\mathbf{M}_{\mathrm{j}} \cdot \mathbf{r}_{\mathrm{ij}})}{r_{ij}^5} - \frac{\mathbf{M}_{\mathrm{i}} \cdot \mathbf{M}_{\mathrm{j}}}{3r_{ij}^3} \right] \right\}$$

where ε is depth of the potential well for the truncated Lennard-Jones potential, R is the radius of the cluster, $\mathbf{r}_{\mathrm{ij}}$ is the distance vector between cluster i and cluster j, r_c is the cut-off radius for the cluster and equals to $2^{7/6}R$, μ_0 is the vacuum permeability. χ is the susceptibility of nanomagnets. $\mathbf{M}_{\mathrm{i}}$ and $\mathbf{M}_{\mathrm{j}}$ are the magnetizations of the nanomagnet clusters i and j. The simulation evolves the system from the totally random initial positions with the magnetization amplitude of the nanomagnet cluster assigned to be 10^7 A m^{-1} at the positive Z direction. During each simulation step, a random cluster will be selected and assigned with a small translational movement of -1.5, 0, and 1.5 μm in X, Y, and Z directions. Additionally, the magnetization of the cluster will be assigned with a random small rotation of -2, 0, and 2 degrees along the X, Y, and Z axes at each step. Each Monte-Carlo step will be accepted if it results in a lower total energy or satisfies the acceptance probability given by $\exp(-\Delta E/k_B T)$. A total of 20,000 steps were performed to allow the system to reach equilibrium.

It is worth mentioning that the value of χ did not influence the whole simulation process because the associated item is a constant regardless of the relative positions of the nanomagnet clusters. In the simulation, we have assigned χ to be equal to 1. For the depth of the potential well ε, we assigned it to be 4.11×10^{-12} J. This value is based on the consideration that the dipole-dipole interaction of each cluster scales with the cube of the cluster radius. For ferrofluid, the typical value of ε is at the level of kT, which is 4.11×10^{-21} J. Since we simulated cluster at the microscale while the ferrofluid is at the nanoscale, ε needs to be amplified by a scaling factor of ${10^3}^3$, which is the cube of the length scale.

Supplementary Note 2. Finite element analysis for the injection process.

The mathematical model used in this analysis was based on the incompressible Navier-Stokes equations with variable density, variable viscosity, surface tension, and gravitation. Fluid flow is controlled by the following equations:

$$\rho\left(\frac{\partial \mathbf{u}}{\partial t} + \mathbf{u} \cdot \nabla \mathbf{u}\right) = -\nabla p + \nabla \cdot \left(\mu \left(\nabla \mathbf{u} + \nabla \mathbf{u}^{T}\right)\right) + \sigma\kappa \mathbf{n} \delta + \rho \mathbf{g} \quad , \quad \nabla \cdot \mathbf{u} = 0 \tag{1}$$

Whereas κ is the interface curvature, δ is the delta function concentrated to the interface, σ is the surface tension coefficient, n is the unit normal to the interface, ρ and μ is the density and viscosity for different fluids. The fluid interface is modeled with the level set function ϕ.

$$\frac{\partial \phi}{\partial t} + \mathbf{u} \cdot \nabla \phi = 0 \tag{2}$$

$$\rho = \rho_{air} + (\rho_{PFM} - \rho_{air})\varphi \tag{3}$$

$$\mu = \mu_{air} + (\mu_{PFM} - \mu_{air})\varphi \tag{4}$$

Supplementary Note 3. Finite element analysis of the reproducibility of the liquid sensor.

Since the liquid disperses differently each time during placement, here we investigate the reproducibility of the shape of the liquid sensor. We conducted simulations to mimic the behavior of liquid sensors dropped at different locations on the skin, observing the variation in dispersion each time. In finite element analysis, the initial position was set at 10 mm. Then, we examined the reproducibility by running simulations with different initial positions of the liquid sensor, ranging from x = 10 mm ± 5 mm. The simulation results are shown in Supplementary Figure 23. When the sensor was placed at positions of 15 mm, 14 mm, 6 mm, and 5 mm, it indeed formed different shapes compared to the initial position (**Supplementary Figure 23a-l**). However, despite these variations, the final formed shapes were very similar to each other (**Supplementary Figure 23c, f, i, l**).

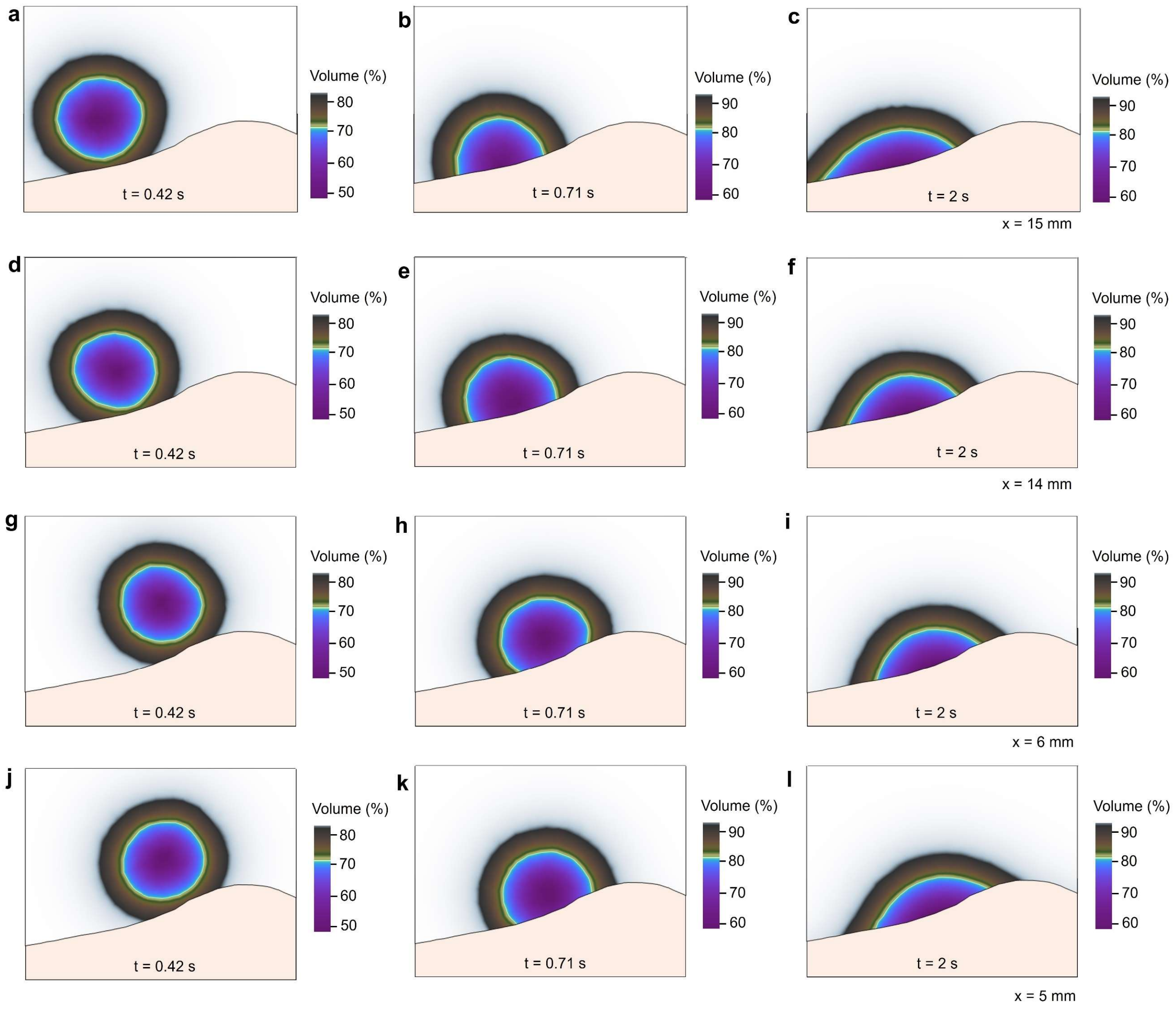

Supplementary Figure 23. **Finite element simulation of the liquid cardiac sensor. a-c**, Finite element simulation of the liquid cardiac sensor applied to the wrist with the initial position set at 15 mm. **d-f**, at 14 mm. **g-i**, at 6 mm. **j-l**, at 5 mm.

In the experiment, we placed liquid sensors with different volumes ranging from 30 µl, 40 µl, and 50 µl of PFM at one position (**Supplementary Figure 24a-c**). We also placed the liquid sensors at different positions (**Supplementary Figure 24d-f**), and the results showed that they formed similar shapes.

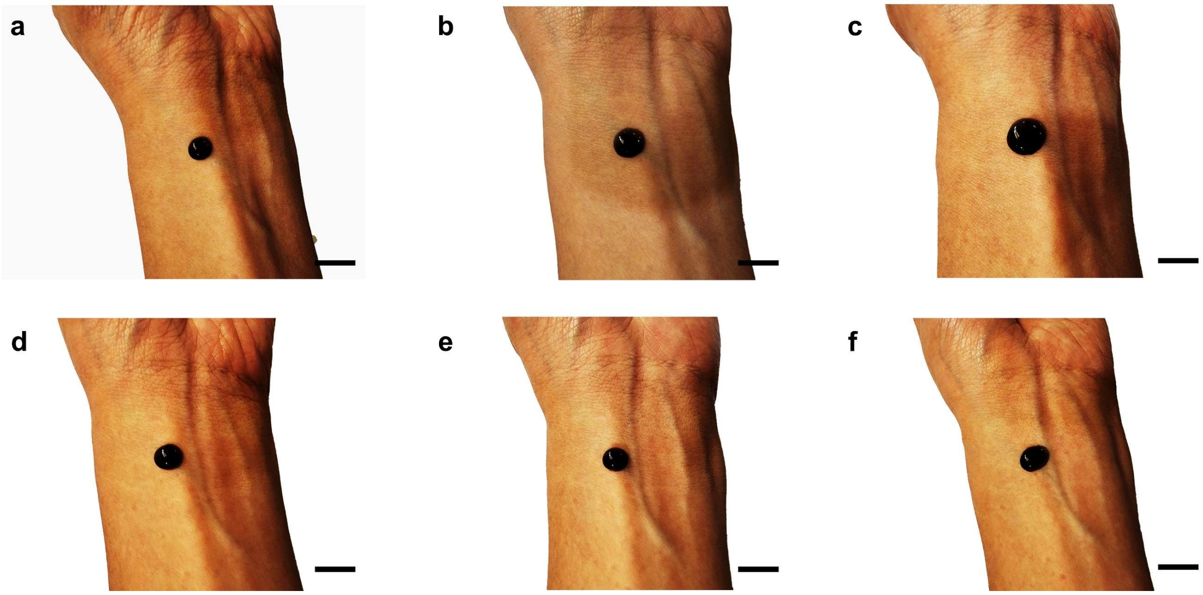

Supplementary Figure 24. Picture of the liquid sensors dispersed on the wrist. The liquid sensors with different volumes ranging from **a**, 30 µl, **b,** 40 µl, and **c,** 50 µl of PFM at one position. The liquid sensors with different locations ranging from **d**, 0 mm, **e**, 1 mm, and **f**, 2 mm. Scale bars, 5 mm. All images were conducted following three independent experiments. Those images yield similar results in different independent experiments. Typical images were shown in the Figure.

Supplementary Note 4. Power consumption of the biosensing board.

The power consumption for the wireless modules is high during the pairing stage, tested to be ~19.8 mA. After the pairing stage, when measuring the waveform continuously, the current for the whole device is tested to be ~6.87 mA. Considering the battery has an output voltage of 3.7 V and a capacity of 380 mAh, we calculate the battery life given its capacity and the output current using the following formula:

$$t = \frac{C}{I} \times (1 - \eta)$$

where t is the battery life, C is the capacity of the battery, I is the output current, η is the derating factor which is set to be 10%. After calculation, the expected time for the liquid sensor is approximately 55.31 hours. Accounting for real-world inefficiencies, the battery is expected to last approximately 49.78 hours.

Supplementary Note 5. Pulse waveform analysis.

We consider pulse wave measurements and analysis as critical non-invasive bioassays for the prognosis and diagnosis of cardiovascular diseases. From pulse wave measurements, several important parameters can be extracted including the PWV, pulse waveform, aortic systolic and diastolic pressures, augmentation index (AIs), and round-trip travel time of the reflecting wave. These extracted parameters can be used to evaluate arterial elasticity and stiffness. Their correlations with cardiovascular risks have been extensively validated in clinical trials[1,2]. Additionally, pulse wave analysis has been recognized as an important supplementary method to blood pressure measurement. Blood pressure in the brachial artery fails to reveal the adverse, atherosclerotic effects of hypertension. Also, the left ventricle is primarily influenced by the pressure in the ascending aorta rather than by the pressure in the brachial artery. Differently, pulse pressure waves measured at the radial artery can be used to evaluate the aortic pressure waveform using a general transfer function[3,4]. Our work has proved that the PFM liquid sensor can precisely measure the radial pulse waveform in ambulatory conditions.

Taking a step further, we have analyzed the obtained pulse wave signals and demonstrated that PWV and SI can be accurately derived from the current signal obtained using PFM (**Supplementary Figure 25**). These findings suggest that the pulse waveforms captured by our PFM liquid sensor encapsulate subtle arterial stiffness information conveyed by the forward and reflected pulse waves. Moreover, the PFM liquid sensor approach offers additional advantages in operation, including minimal training requirements and the elimination of the need for calibration, which is necessary in application tonometry. We believe that PFM liquid sensors will bring a transformative impact to the field of pulse wave analysis, offering promising applications in predicting cardiovascular diseases and screening cardiovascular treatments.

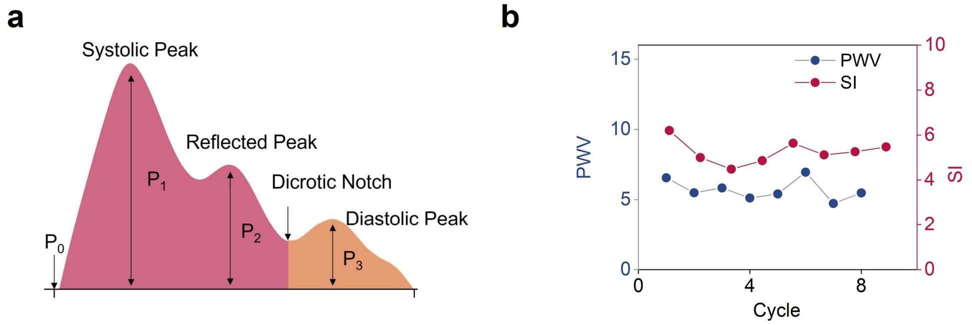

Supplementary Figure 25. **Typical pulse wave profile in one cardiac cycle obtaining. a**, Typical pulse wave profile in one cardiac cycle obtaining. **b**, Measured PWV and SI.

Supplementary Table 1. Parameters of the magnetic materials and airflow in the simulation.

Media	Density	Viscosity	Surface tension
PFM	2.3×10^3 kg/m^3	5 Pa·s	0.079 N/m
Air	1.22 kg/m^3	1.81×10^{-5} Pa·s	N/A
