# Supplementary material for: A reconfigurable and conformal liquid sensor for ambulatory cardiac monitoring
Source: Nat Commun. 2024 Oct 1;15:8492. doi: 10.1038/s41467-024-52462-8 (PMC11445489; doi:10.1038/s41467-024-52462-8)
Supplement: Supplementary file 3 — Reporting Summary [file 41467_2024_52462_MOESM3_ESM.pdf]

nature portfolio

Corresponding author(s): Jun Chen

Last updated by author(s): Aug 24, 2024

Reporting Summary

Nature Portfolio wishes to improve the reproducibility of the work that we publish. This form provides structure for consistency and transparency in reporting. For further information on Nature Portfolio policies, see our Editorial Policies and the Editorial Policy Checklist.

Statistics

For all statistical analyses, confirm that the following items are present in the figure legend, table legend, main text, or Methods section.

n/a	Confirmed	
☐	☒	The exact sample size (*n*) for each experimental group/condition, given as a discrete number and unit of measurement
☐	☒	A statement on whether measurements were taken from distinct samples or whether the same sample was measured repeatedly
☐	☒	The statistical test(s) used AND whether they are one- or two-sided<br>*Only common tests should be described solely by name; describe more complex techniques in the Methods section.*
☐	☒	A description of all covariates tested
☐	☒	A description of any assumptions or corrections, such as tests of normality and adjustment for multiple comparisons
☐	☒	A full description of the statistical parameters including central tendency (e.g. means) or other basic estimates (e.g. regression coefficient) AND variation (e.g. standard deviation) or associated estimates of uncertainty (e.g. confidence intervals)
☒	☐	For null hypothesis testing, the test statistic (e.g. *F*, *t*, *r*) with confidence intervals, effect sizes, degrees of freedom and *P* value noted<br>*Give P values as exact values whenever suitable.*
☒	☐	For Bayesian analysis, information on the choice of priors and Markov chain Monte Carlo settings
☒	☐	For hierarchical and complex designs, identification of the appropriate level for tests and full reporting of outcomes
☒	☐	Estimates of effect sizes (e.g. Cohen's *d*, Pearson's *r*), indicating how they were calculated

Our web collection on statistics for biologists contains articles on many of the points above.

Software and code

Policy information about availability of computer code

Data collection	Data collection was performed using a Zeiss Axio Observer Z1 inverted fluorescence microscope and Leica SP8-STED/FLIM/FCS confocal microscope for fluorescence imaging; magnetic particle size distribution was determined by ZEISS Supra 40VP and TF20 high-resolution electron microscopy; the electrical signal were recored by LabVIEW 2019; the magnetic hysteresis loops were measured by a SQUID magnetometer Quantum Design, MPMS 3. The magnetic flux density was measured with MLX 90393 Melexis. Python 3.7 was used to record all the data simultaneously.
Data analysis	Data analysis was performed by free or commercial software platforms, including ImageJ 1.53t. Origin 2018 was used to generate bar charts or box charts and perform statistical analysis. Matlab R2022b was used for the Monte Carlo simulation of the oriented and ramified nanomagnet network.

For manuscripts utilizing custom algorithms or software that are central to the research but not yet described in published literature, software must be made available to editors and reviewers. We strongly encourage code deposition in a community repository (e.g. GitHub). See the Nature Portfolio guidelines for submitting code & software for further information.

Data

Policy information about availability of data

All manuscripts must include a data availability statement. This statement should provide the following information, where applicable:

- Accession codes, unique identifiers, or web links for publicly available datasets
- A description of any restrictions on data availability
- For clinical datasets or third party data, please ensure that the statement adheres to our policy

All data supporting the findings of this study are available within the article and its supplementary files. Any additional requests for information can be directed to, and will be fulfilled by, the corresponding authors. Source data are provided with this paper.

Research involving human participants, their data, or biological material

Policy information about studies with human participants or human data. See also policy information about sex, gender (identity/presentation), and sexual orientation and race, ethnicity and racism.

Reporting on sex and gender	3 male participants were recruited for the experiment to test device performance.
Reporting on race, ethnicity, or other socially relevant groupings	Gender and other biographical information were irrelevant for the human study conducted in our experiment.
Population characteristics	The human participants in this study are students at University of California, Los Angeles with the age from 20 - 30 years old. Population characteristics are not relevant to the experiment or the results of this study.
Recruitment	Participants were recruited from the Department of Bioengineering at University of California, Los Angeles.
Ethics oversight	The sensor was used on human subjects in compliance with all the ethical regulations under a protocol (ID: 20-001882) that was approved by the Institutional Review Board (IRB) at University of California, Los Angeles. All participating subjects were affiliated with the University of California, Los Angeles, and were provided informed consent before participation in the study.

Note that full information on the approval of the study protocol must also be provided in the manuscript.

Field-specific reporting

Please select the one below that is the best fit for your research. If you are not sure, read the appropriate sections before making your selection.

☒ Life sciences ☐ Behavioural & social sciences ☐ Ecological, evolutionary & environmental sciences

For a reference copy of the document with all sections, see nature.com/documents/nr-reporting-summary-flat.pdf

Life sciences study design

All studies must disclose on these points even when the disclosure is negative.

Sample size	A minimum of three independent experiments were carried out for all studies. The sample size necessary to detect a significant effect was estimated by using data from pilot studies. Statistical analysis was performed using Origin 2018 software. A p-value < 0.05 was considered significant.
Data exclusions	No data exclusion in this research
Replication	In all experiments, either replicate or triplicate of samples were processed in parallel. All experiments were performed independently 3 times or more and consistent results were evident across independent experiments.
Randomization	n/a
Blinding	All data were acquired and analyzed by softwares with objective standard, so blinding is not relevant to the data analysis.

Reporting for specific materials, systems and methods

We require information from authors about some types of materials, experimental systems and methods used in many studies. Here, indicate whether each material, system or method listed is relevant to your study. If you are not sure if a list item applies to your research, read the appropriate section before selecting a response.

Materials & experimental systems

n/a	Involved in the study
☒	☐ Antibodies
☒	☐ Eukaryotic cell lines
☒	☐ Palaeontology and archaeology
☒	☐ Animals and other organisms
☒	☐ Clinical data
☒	☐ Dual use research of concern
☒	☐ Plants

Methods

n/a	Involved in the study
☒	☐ ChIP-seq
☒	☐ Flow cytometry
☒	☐ MRI-based neuroimaging

Plants

Seed stocks	n/a
Novel plant genotypes	n/a
Authentication	n/a
